# Case Report: Transient hypertension and myalgia following mavacamten therapy in a patient with hypertrophic obstructive cardiomyopathy

**DOI:** 10.3389/fcvm.2025.1578356

**Published:** 2025-09-10

**Authors:** Mengling Peng, Yu Fu, Cong Qin, Shanshan Zhou, Jian Sun

**Affiliations:** The Center of Cardiovascular Diseases, The First Hospital of Jilin University, Changchun, China

**Keywords:** hypertrophic cardiomyopathy, mavacamten, hypertension, myalgia, hemodynamic adaptation

## Abstract

**Background:**

Mavacamten has been demonstrated to be effective in the treatment of hypertrophic obstructive cardiomyopathy (HOCM). However, its hemodynamic impact and extracardiac effects require further characterization.

**Case presentation:**

We report a case of a 68-year-old female diagnosed with severe HOCM who experienced transient hypertension (165/105 mmHg) and myalgia four weeks after mavacamten initiation. Despite a significant reduction in LVOT obstruction (from 64 mmHg–18 mmHg) and an increase in LVEF to 78%, the patient exhibited a transient hypertensive response that resolved spontaneously within two weeks without intervention. Myalgia was present without corresponding elevations in serum creatine kinase.

**Conclusions:**

This case highlights a previously unrecognized transient hypertensive phase associated with myosin inhibition, potentially related to ventriculo-arterial decoupling and peripheral vascular adaptation. Additionally, the dissociation between myalgia and CK elevation suggests alternative skeletal muscle involvement mechanisms. Close blood pressure monitoring and further investigation into the extracardiac effects of mavacamten are warranted.

## Case details

This report presents the case of a 68-year-old female diagnosed with severe hypertrophic obstructive cardiomyopathy (HOCM). The patient presented with exertional chest discomfort, which occurred during physical activity and was relieved by rest. She denied associated symptoms such as palpitations, syncope, or dyspnea. There was no history of hypertension, non-sustained ventricular tachycardia, syncope, or a family history of sudden cardiac death. Upon admission, her blood pressure was 142/63 mmHg, which was a single measurement obtained during the initial visit and did not reflect persistent hypertension. Her heart rate was 69 beats per minute, and physical examination revealed a systolic ejection murmur at the third left intercostal space along the sternal border.

**Figure 1:**
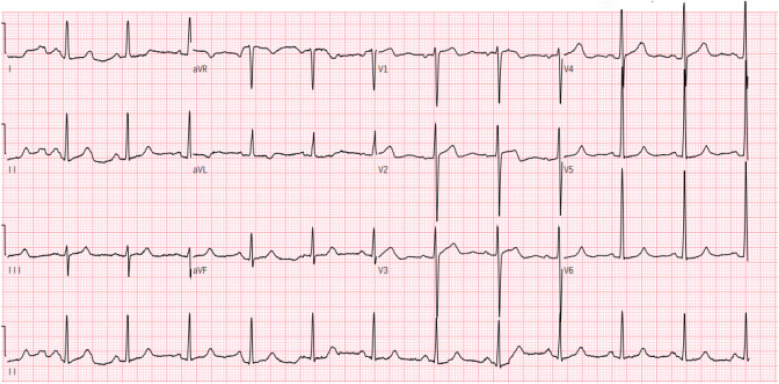
Electrocardiogram upon admission.

**Figure 2:**
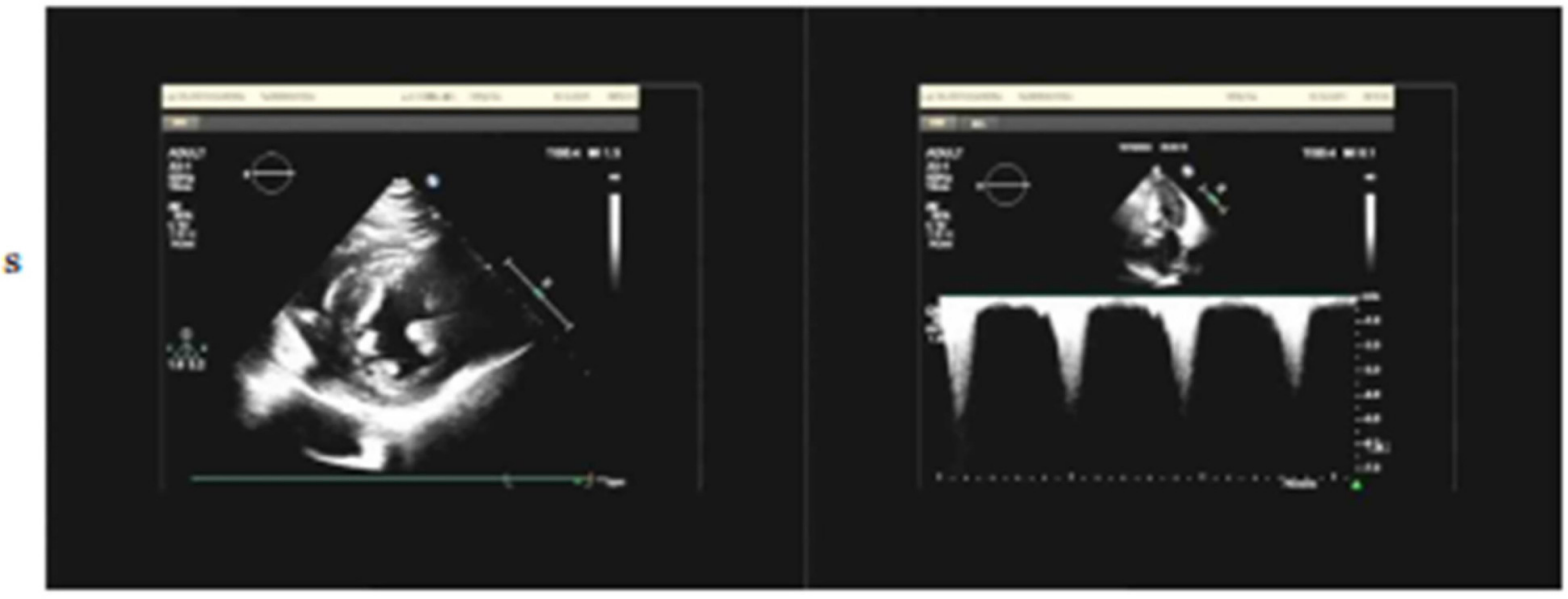
Echocardiogram upon admission.

Electrocardiography (ECG) demonstrated sinus rhythm with left ventricular hypertrophy and ST-T wave changes (Figure 1). Routine laboratory tests, including coagulation and renal function assessments, were within normal ranges, except for mild liver enzyme elevation (AST: 54.6 U/L; ALT: 59.1 U/L). The patient's brain natriuretic peptide (BNP) level was elevated at 249.08 pg/ml,serum creatinine (48.3 μmol/L), troponin (0.03 ng/ml), TSH (2.596 uIU/ml), FT3 (3.69 pmol/L), and FT4 (12.77 pmol/L), all within normal ranges. Transthoracic echocardiography (TTE) revealed asymmetric left ventricular hypertrophy, predominantly affecting the mid-septum, with a maximal thickness of 17 mm. The systolic anterior motion (SAM) of the mitral valve and subvalvular apparatus was present, contributing to increased LVOT blood flow velocity. The resting peak LVOT velocity was 2.99 m/s (pressure gradient 36 mmHg), increasing to 4.07 m/s (pressure gradient 66 mmHg) after Valsalva maneuver. The left ventricular ejection fraction (LVEF) was 64%, with evidence of diastolic dysfunction (E/A ratio of 1.13, E/e′ > 14) and left atrial enlargement (40 mm) (Figure 2).

**Figure 3:**
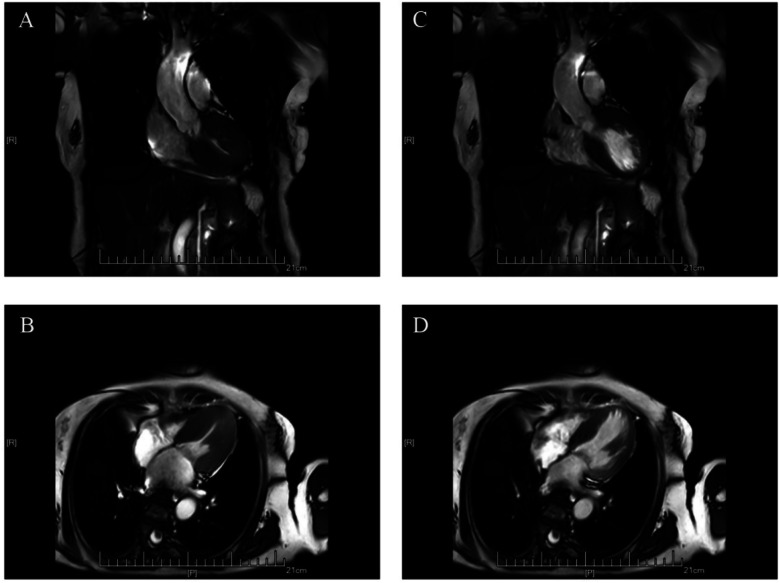
Cardiac MRI scans showing hypertrophic cardiomyopathy. **(A,C)** Coronal views; **(B,D)** Axial views.

**Figure 4:**
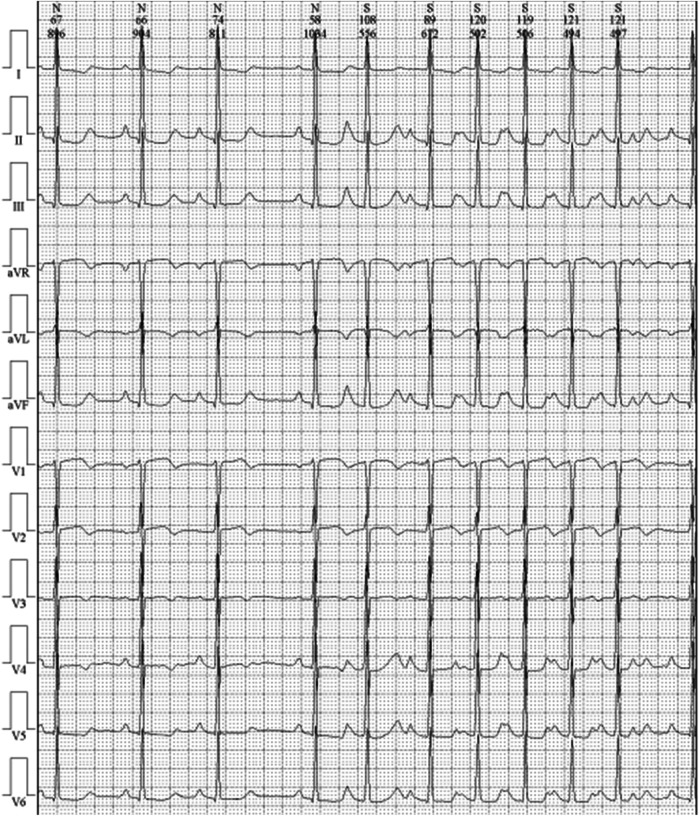
24 h ambulatory electrocardiogram upon admission.

Cardiac magnetic resonance imaging (CMR) demonstrated patchy mid-septal delayed gadolinium enhancement without abnormal myocardial perfusion (Figure 3). A 24 h ambulatory ECG (Holter) revealed occasional ventricular and atrial premature beats, as well as brief episodes of atrial tachycardia (Figure 4). Coronary angiography identified scattered plaques in the proximal left anterior descending artery (LAD) with a maximal stenosis of 30% and approximately 20% stenosis in the mid-proximal right coronary artery (RCA), without significant obstruction in other coronary segments.

The patient was eventually diagnosed with HOCM (NYHA Class II) and initiated on mavacamten therapy, alongside a low-dose beta-blocker (bisoprolol 1.25 mg daily). Following mavacamten initiation, the patient reported significant symptomatic improvement in chest discomfort. However, four weeks post-treatment, she developed transient hypertension (165/105 mmHg), though she remained asymptomatic. No antihypertensive intervention was administered, and blood pressure spontaneously decreased to 150/90 mmHg within two days and gradually normalized to 130/80 mmHg over the following 2 weeks.The patient declined further evaluation for secondary causes of hypertension. Two months after starting mavacamten, repeat echocardiography showed a reduced LVOT gradient (18 mmHg at rest, 19 mmHg post-Valsalva maneuver), an increased LVEF of 78%, and a ventricular fractional shortening (FS) of 46%. The maximum septal thickness remained 17 mm. The observed increase in LVEF was considered to reflect improved ventricular outflow dynamics due to LVOT gradient relief rather than true augmentation of myocardial contractility.

Additionally, the patient reported new-onset back pain six weeks after initiating mavacamten therapy. Serum creatine kinase (CK) levels were within normal limits, and clinical evaluation did not reveal signs suggestive of musculoskeletal, renal, or neurological involvement. Common alternative causes of back pain in elderly patients, including degenerative spine disease, musculoskeletal strain, and osteoporotic fracture, were considered less likely in this case due to the absence of localized tenderness, normal neurological examination, and normal CK levels. Although the exact etiology of the back pain could not be determined, a potential drug-related effect could not be excluded.

## Discussion

The patient developed a transient elevation in blood pressure after initiating mavacamten therapy. She had no prior history of hypertension in outpatient records from multiple institutions. Although secondary causes of hypertension (such as renal artery stenosis, primary aldosteronism, and pheochromocytoma) were not evaluated due to patient refusal, the transient nature and spontaneous resolution of the episode support a treatment-related etiology. The absence of pre-treatment home or ambulatory blood pressure monitoring is acknowledged as a limitation in this case.

We hypothesize that in the early phase of myosin inhibition, reduction of the LVOT gradient precedes the full development of negative inotropic effects. This sudden relief of outflow obstruction may transiently increase peripheral vascular resistance, potentially through neurohormonal activation and exposure of underlying arterial stiffness, before subsequent stabilization as myosin inhibition reaches steady state and myocardial contractility and filling pressures decline. A similar hemodynamic phenomenon has been described after transcatheter aortic valve implantation (TAVI), where abrupt afterload reduction leads to a short-lived rise in blood pressure before hemodynamic equilibrium is restored. Although this analogy is physiologically plausible, the absence of invasive hemodynamic or vascular stiffness measurements in our case limits definitive mechanistic confirmation. However, we acknowledge that in the absence of invasive hemodynamic measurements or vascular stiffness assessments, our explanation remains speculative.

Wang et al. reported in a *post hoc* analysis of the EXPLORER-HCM study that hypertensive patients receiving mavacamten had significantly higher blood pressure compared to placebo (130 ± 14.8 vs. 125 ± 15.3 mmHg, *P* < 0.01) (1). Similarly, in the SEQUOIA-HCM study (2), patients treated with aficamten—a myosin inhibitor with a shorter half-life—demonstrated a higher incidence of hypertension compared with the placebo group (7.7% vs. 2.1%). A recent case report by Maurizi et al. (3) described a patient with obstructive HCM who developed sustained hypertension 10 days after mavacamten initiation, requiring intensified antihypertensive management.

The earlier occurrence of transient hypertension in our patient compared to findings from EXPLORER-HCM and SEQUOIA-HCM may reflect several factors. First, differences in blood pressure monitoring practices likely played a role; in this case, frequent outpatient visits and home BP measurements enabled early detection of short-term fluctuations, whereas clinical trial protocols typically assess BP at scheduled study visits, potentially overlooking transient changes. Second, patient-specific characteristics such as advanced age, postmenopausal status, and increased arterial stiffness may have predisposed this individual to more pronounced vascular responses during the initial hemodynamic adjustment to LVOT gradient reduction. Lastly, the rapid decrease in LVOT obstruction and associated alterations in cardiac output in real-world settings might differ from the more gradual changes observed under the controlled titration protocols of clinical trials, leading to earlier hemodynamic shifts.

While transient hypertension following TAVI has been associated with improved left ventricular function and favorable clinical outcomes in the short and long term (4–7), the clinical implications of transient hypertension following mavacamten therapy remain unclear. It is unknown whether such blood pressure fluctuations signal a favorable ventricular remodeling response or indicate a need for closer hemodynamic monitoring.Prospective studies with systematic BP phenotyping and vascular stiffness measurements are needed to clarify prognostic significance.

The patient also experienced back pain following mavacamten initiation, without concomitant CK elevation. While myalgia is an uncommon adverse effect of mavacamten, its pathophysiology remains poorly understood. Unlike statin-induced myopathy, which involves direct myotoxicity and mitochondrial dysfunction (8), myosin inhibitors act through allosteric modulation of β-cardiac myosin, altering cross-bridge cycling without directly affecting skeletal muscle metabolism (9). However, preclinical studies have shown that mavacamten exhibits approximately 4 fold lower selectivity for cardiac myosin over skeletal muscle myosin, and at higher concentrations, it can inhibit skeletal muscle myosin ATPase activity *in vitro* (9, 10). Although the clinical relevance of these findings remains uncertain, they raise the possibility that off-target effects on skeletal muscle myosin could contribute to musculoskeletal symptoms, such as transient fatigue or discomfort, in some patients. Further studies are warranted to explore these potential mechanisms.

Emerging pharmacovigilance data suggest that myosin inhibitors such as mavacamten may be associated with novel and unexpected adverse events. A recent analysis highlighted occurrences of urinary tract infections, gout flares, and peripheral edema in patients receiving mavacamten (11). While our patient did not develop these specific events, awareness of these potential risks is important for clinicians, and routine monitoring should include vigilance for such symptoms, particularly in elderly patients or those with pre-existing risk factors.

Furthermore, the differential diagnosis of hypertrophic cardiomyopathy (HCM) must be carefully considered to avoid misdiagnosis. Fabry disease, a lysosomal storage disorder, can present with concentric or asymmetric left ventricular hypertrophy and elevated cardiac biomarkers but was considered unlikely in this elderly female due to absence of extracardiac manifestations such as angiokeratomas, corneal verticillata, or proteinuria (12). Cardiac amyloidosis, particularly transthyretin amyloidosis, can mimic HCM in elderly patients; however, echocardiography and CMR in this case did not show characteristic features such as apical sparing on strain imaging or diffuse subendocardial late gadolinium enhancement (13). Athlete's heart was excluded based on the patient's age, sedentary lifestyle, and lack of significant physiological hypertrophy regression on detraining (14). Hypertensive heart disease was unlikely given the patient's normotensive history and absence of global concentric hypertrophy (15).

The relationship between HCM and exercise tolerance remains an area of active investigation. Current consensus supports individualized exercise recommendations balancing the benefits of moderate aerobic activity against the risks of arrhythmias and sudden cardiac death. Mavacamten therapy may improve exercise capacity by reducing LVOT obstruction and enhancing diastolic filling. Our sedentary patient was advised to engage in light-to-moderate physical activity under medical supervision. Further studies are needed to define exercise guidelines tailored to patients on myosin inhibitors.

In conclusion, given the transient hypertensive response observed in this case, regular blood pressure monitoring should be emphasized in clinical practice. Larger prospective and registry-based studies are warranted to determine whether transient hypertension observed during mavacamten initiation represents a consistent phenomenon and whether it has any impact on long-term clinical outcomes in patients with HOCM.

## Data Availability

The original contributions presented in the study are included in the article/Supplementary Material, further inquiries can be directed to the corresponding authors.
